# The clinical characteristics of patients with congenital nephrotic syndrome secondary to *NPHS1* mutation: Is nephrectomy still a therapeutic option for selected cases?

**DOI:** 10.1007/s00467-025-06774-6

**Published:** 2025-04-23

**Authors:** Yüksel Uğurlu, Bora Gülhan, İsmail Dursun, Hülya Nalçacıoğlu, Gülşah Kaya Aksoy, Nur Canpolat, Aysun Bayazıt, Zeynep Birsin Özçakar, Selcuk Yüksel, Gönül Parmaksız, Gülşah Özdemir, Eda Didem Kurt-Şükür, Ali Düzova, Mutlu Hayran, Fatih Ozaltin

**Affiliations:** 1 Department of Pediatrics, Faculty of Medicine, Hacettepe University, Ankara, Türkiye; 2 Department of Pediatric Nephrology, Faculty of Medicine, Hacettepe University, Sihhiye, 06100 Ankara, Türkiye; 3 Department of Pediatric Nephrology, Faculty of Medicine, Erciyes University, Kayseri, Türkiye; 4 Department of Pediatric Nephrology, Faculty of Medicine, Ondokuz Mayıs University, Samsun, Türkiye; 5 Department of Pediatric Nephrology, Faculty of Medicine, Akdeniz University, Antalya, Türkiye; 6 Department of Pediatric Nephrology, Istanbul University-Cerrahpasa, Cerrahpasa Faculty of Medicine, Istanbul, Türkiye; 7 Department of Pediatric Nephrology, Faculty of Medicine, Çukurova University, Adana, Türkiye; 8 Department of Pediatric Nephrology, Faculty of Medicine, Ankara University, Ankara, Türkiye; 9 Department of Pediatric Nephrology, Faculty of Medicine, ÇAnakkale Onsekiz Mart University, Çanakkale, Türkiye; 10 Department of Pediatric Nephrology, Başkent University Adana Dr. Turgut Noyan Training and Research Center, Adana, Türkiye; 11 Department of Preventive Oncology, Hacettepe University Cancer Institute, Ankara, Türkiye; 12 Nephrogenetics Laboratory, Department of Pediatric Nephrology, Faculty of Medicine, Hacettepe University, Ankara, Türkiye; 13 Center for Genomics and Rare Diseases, Hacettepe University, Ankara, Türkiye; 14 Department of Bioinformatics, Hacettepe University Institute of Health Sciences, Ankara, Türkiye

**Keywords:** Congenital nephrotic syndrome, *NPHS1* mutation, Nephrin, Nephrectomy

## Abstract

**Background:**

Managing congenital nephrotic syndrome (CNS) remains a clinical challenge. While albumin infusions and nephrectomy have been long-standing treatments, a conservative approach is increasingly favored. This study aimed to compare clinical outcomes between nephrectomy (Nx) and non-Nx in patients with bi-allelic *NPHS1* mutations.

**Methods:**

This retrospective cohort study included 29 pediatric CNS patients (15 female, 14 male) with confirmed *NPHS1* mutations. Clinical parameters including albumin infusion requirements, infections, hospitalizations, growth, and survival rates were analyzed in the Nx and non-Nx groups.

**Results:**

The median age at the time CNS was diagnosed was 29 days (IQR: 11–62 days). In all, 24 patients (82.8%) had homozygous *NPHS1* mutations and 5 (17.2%) had compound heterozygous *NPHS1* mutations. None of the patients had Fin-major mutation (i.e., p. Leu41 Aspfs*50*)*. Unilateral/bilateral nephrectomy was performed in 16 patients. At 12 months post-nephrectomy the number of albumin infusions required, infections, and hospitalizations decreased significantly in the Nx group, as compared to the pre-nephrectomy period (*p* = 0.001, *p* = 0.027, and *p* = 0.004, respectively). Among the 13 (44.8%) patients in the non-Nx group, at 12 months after CNS was diagnosed the number of serum albumin infusions required significantly decreased (*p* = 0.007); however, the number of infections and hospitalization did not differ significantly (*p* = 0.589 and *p* = 0.5, respectively). Receiver operating characteristic (ROC) analysis showed that requiring albumin infusions ≥ 14 days/month predicted the decision to perform nephrectomy with 68% accuracy (73% sensitivity and 62% specificity).

**Conclusions:**

Nephrectomy reduces albumin infusions, infections, and hospitalizations, suggesting it may be a beneficial treatment for selected CNS patients with *NPHS1* mutations.

**Graphical abstract:**

**Figure:**
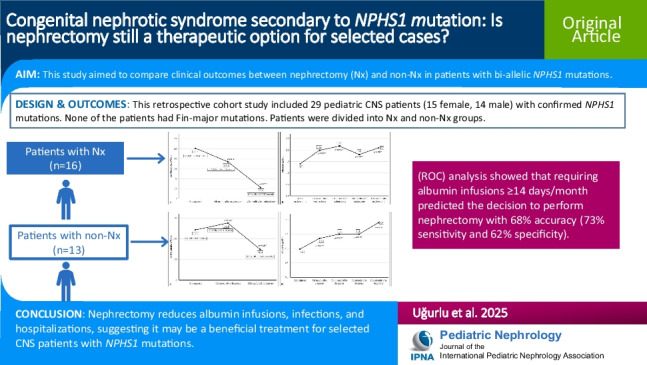
A higher resolution version of the Graphical abstract is available as Supplementary information

**Supplementary Information:**

The online version contains supplementary material available at 10.1007/s00467-025-06774-6.

## Introduction

Congenital nephrotic syndrome (CNS) is a glomerular disorder characterized by significant protein loss from the kidneys. The disease usually begins during the antenatal period, and most patients are diagnosed during the 3 months following birth [1, 2]. The loss of essential proteins, including thyroid-binding globulin, antithrombin III, immunoglobulin G, complement components C3 and C5, as well as factors B and D, can result in serious complications. These complications include hypothyroidism, thrombosis, and a heightened risk of infections, with sepsis being the leading cause of death in these patients [3–6].

The management of CNS focuses on maintaining intravascular volume, controlling edema, supporting healthy growth and development, and preventing life-threatening complications until kidney transplantation [2]. Optimal management of CNS patients is both challenging and subject to debate. Spontaneous remission is extremely rare, and the use of steroids or other immunosuppressive drugs is not recommended. Treatment typically involves serum albumin infusions, reducing proteinuria with angiotensin-converting enzyme inhibitors (ACEi) and/or indomethacin. Over the past 40 years, an aggressive treatment approach, including bilateral nephrectomy, has been employed to decrease the severity of proteinuria; however, more recent findings show that nephrectomy and more conservative approaches without nephrectomy yield similar outcomes [2].

Relevant research has focused on CNS patients with mutations in the *NPHS1, NPHS2, WT1*, or *LAMB2* genes [7, 8]. Although CNS presents as a shared phenotype, each genetic abnormality can lead to a unique disease course and prognosis [9]. As a result, collective analyses that overlook the underlying genetic etiology of CNS might not provide an accurate perspective. The present study aimed to determine if nephrectomy is associated with better clinical outcomes in patients with bi-allelic *NPHS1* mutations, as compared to those managed conservatively. This nationwide Turkish collaborative study analyzed data collected exclusively from CNS patients with bi-allelic *NPHS1* mutations that underwent nephrectomy or were managed conservatively without nephrectomy.

## Methods

### Definitions and data collection

This retrospective longitudinal cohort study included 29 pediatric CNS patients with genetically confirmed bi-allelic *NPHS1* mutations that were followed-up at 9 pediatric nephrology departments across Türkiye between 2005 and 2023. Standardized data collection forms were used for consistency. The study protocol was approved by the Hacettepe University Ethics Committee (2022/05–29).

We assessed data including serum albumin and creatinine levels, as well as height and body weight in all patients at the time CNS was diagnosed. In the nephrectomy group (Nx group) these parameters were also evaluated at 1, 3, 6, and 12 months post-nephrectomy and at the last visit. Similarly, in the conservatively treated group (non-Nx group) the parameters were assessed at 1, 3, 6, and 12 months after the diagnosis of CNS and at the last visit. We also analyzed the number of albumin infusions administered each month (day number per month). Albumin infusions were administered to patients with clinical signs such as anasarca, reduced urine output, and/or respiratory distress, particularly when serum albumin levels were below 2 g/dL. In all cases, intravenous furosemide was administered following albumin infusion to enhance diuresis. In the Nx group the number of albumin infusions was evaluated both before and after nephrectomy, whereas in the non-Nx group infusions were assessed prior to the start of medical treatment and again 1 year after its initiation. Additionally, the number of infections and hospitalizations within 12 months post-CNS diagnosis were compared between the 2 groups.

The estimated glomerular filtration rate (eGFR) was calculated using the modified Schwartz formula, considering patient age, height, and body weight [10]. Chronic kidney disease (CKD) stages were classified according to KDIGO guidelines based on eGFR values, as follows [11]: 0–14 ml/min/1.73 m^2^ as stage 5; 15–29 mL/min/1.73 m^2^ as stage 4; 30–59 mL/min/1.73 m^2^ as stage 3; 60–89 mL/min/1.73 m^2^ as stage 2; > 90 mL/min/1.73 m^2^ as stage 1 or normal. Patient z-scores for height and body weight were also evaluated, calculated according to World Health Organization (WHO) standards, as recommended by the Turkish Society of Pediatric Endocrinology and Diabetes (www.ceddcozum.com). Additionally, the results of *NPHS1* mutation analysis were obtained from patient records. All patients underwent mutation analysis using a next-generation sequencing panel composed of the following genes: *ACTN4, C3, CD46 (MCP),CFH, CFB, CFI, CFHR1, CFHR2, CFHR3, CFHR4, CFHR5, COL4 A3, COL4 A4, COL4 A5, COQ2, COQ6, COQ8B, DGKE, EMP2, FAN1, GLA, INF2, LAGE3, LAMB2,LMX1B, MYO1E, NPHS1, NPHS2, NXF5, NUP93, NUP205, OSGEP, PDSS2, PLCε1, PLG, PTPRO, SCARB2, SGPL1, SMARCAL1, THBD, TRPC6, TPRKB, TP53RK, TTC21B, XPO5,* and *WT1*.

### Statistical analysis

Data were analyzed using IBM SPSS Statistics for Windows v.22.0 (IBM Corp., Armonk, NY). Baseline patient demographics and clinical features were summarized using descriptive statistical methods and are shown as mean ± SD or median with interquartile range (IQR, 25 th- 75 th percentiles). Frequency tables were used to visualize categorical data. For parametric data, unpaired t-tests were used to compare mean values, whereas Fisher’s exact test was used to analyze categorical group differences. The Kruskal–Wallis test and post-hoc Mann–Whitney U test with Bonferroni adjustment were used to evaluate differences between the 2 patient groups. Receiver operating characteristic (ROC) analysis was also performed to determine the cut-off value for the number of albumin infusions. The level of statistical significance was set at p < 0.05.

## Results

### Patient characteristics

The study included 29 patients (15 female and 14 male). The consanguinity rate among the cohort was 58.6%. The median age at the time CNS was diagnosed was 29 days (IQR: 11–62). The median serum albumin and creatinine levels, and eGFR at the time of CNS diagnosis were 1.0 g/dL (IQR: 0.8–1.5), 0.17 mg/dL (IQR: 0.1–0.3), and 121 mL/min/1.73 m^2^ (IQR: 70.2–194.1), respectively. At the time of CNS diagnosis 15 patients (65.2%) were classified as stage 1 CKD, 3 (13%) as stage 2 CKD, 3 (13%) as stage 3 CKD, 1 (4.3%) as stage 4 CKD, and 1 (4.3%) as stage 5 CKD.

Among all patients, the median number of infections and hospitalizations at 12 months post-diagnosis of CNS were 3 (IQR: 1–4) and 4 (IQR: 2–6), respectively. All patients received ACEi, whereas indomethacin was administered to 6 (20.6%) patients. At the time CNS was diagnosed the median number of albumin infusions was 16 days/month (IQR: 12–28), with a median albumin dose of 2 g/kg/day (IQR: 1–3). Levothyroxine and statins were administered to 17 (58.6%) patients and 4 (13.8%) patients, respectively. Anticoagulant or antithrombotic prophylaxis was administered to 13 (44.8%) patients, and central venous catheters were placed in 21 (72.4%) patients. Antibiotic prophylaxis was given to 4 (13.8%) patients and antiviral prophylaxis was given to 2 (6.9%) patients. During follow-up 16 (55%) patients underwent unilateral or bilateral nephrectomy (Nx group) and 13 (45%) patients were managed conservatively without nephrectomy (non-Nx group).

### Genetic data

**Fig. 1:**
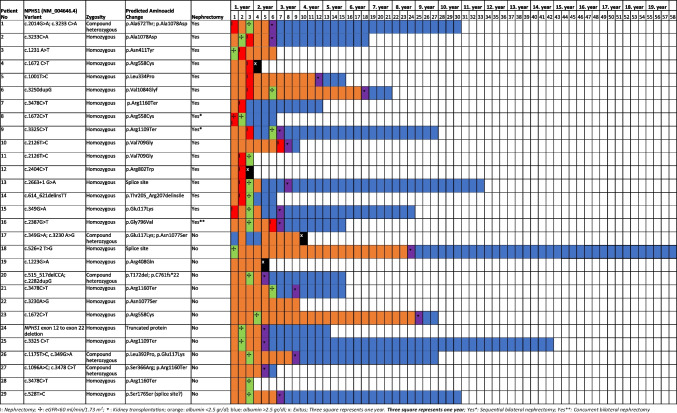
Genetic analysis and clinical outcome of the patients

Among the 29 patients, 24 (82.8%) had homozygous *NPHS1* mutations and 5 (17.2%) had compound heterozygous *NPHS1* mutations. None of the patients carried the Fin-major mutation (p.Leu41 Aspfs*50*)*; however, 2 patients—1 in the Nx group and 1 in the non-Nx group—had the Fin-minor mutation (p.Arg1109*). The most common mutation was NM_004646.4 c.3478 C > T (p.Arg1160 Ter), which was noted in 10.3% of the patients (Fig. 1).

### The Nx group

In all, 13 patients (81.3%) underwent unilateral nephrectomy and 3 patients (18.7%) underwent bilateral nephrectomy. The median age at the time of nephrectomy was 6.5 months (IQR: 4.25–11.75). Among the 3 bilateral nephrectomy patients, 1 underwent the procedure at age 21 months and the other 2 had sequential nephrectomies (at age 4 and 17 months [*n* = 1], and at 6 and 17 months [*n* = 1]). At the time of nephrectomy the median serum albumin and creatinine levels, and eGFR were 1.4 g/dL (IQR: 1.0–1.9), 0.2 mg/dL (IQR: 0.16–0.3), and 101.2 mL/min/1.73 m^2^ (IQR: 70.2–371), respectively.

**Fig. 2:**
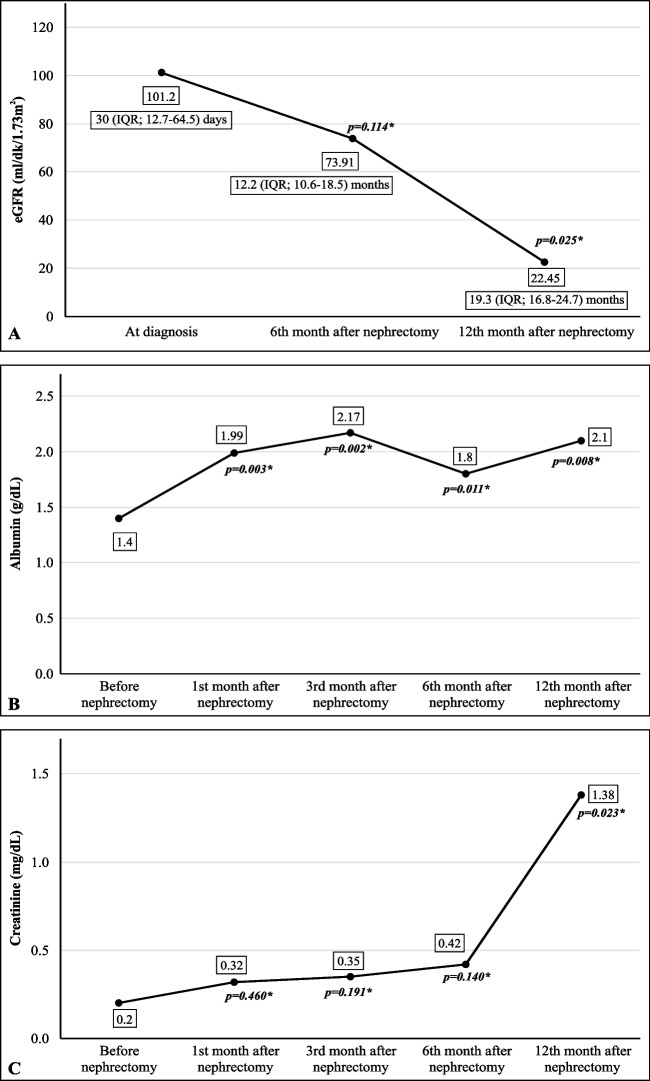
eGFR (**A**), the serum albumin level (**B**), and the serum creatinine level (**C**) before and after nephrectomy (*n* = 16)

CKD staging at the time of nephrectomy was as follows: stage 1: *n* = 7 (63.6%); stage 2: *n* = 2 (18.2%); and stage 3: *n* = 2 (18.2%). The median number of albumin infusions at the time of nephrectomy was 20 days/month (IQR: 12–28). Following nephrectomy, the serum albumin and creatinine levels increased (*p* = 0.008 and *p* = 0.023, respectively) whereas the eGFR decreased (*p* = 0.025). This trend remained consistent even after excluding patients who underwent bilateral nephrectomy (Fig. 2).

Before nephrectomy, the median number of albumin infusions per month (day/month), the median number of infections and hospitalizations per year, and median hospitalization duration (days) were 20 (IQR: 12–28), 3 (IQR: 2–4), 4 (IQR: 2.25–6), and 13 (IQR: 11–27.6), respectively. At 12 months post-nephrectomy these values significantly decreased to 8 (IQR: 1–12), 2 (IQR: 0.75–3), 2 (IQR: 0–3), and 6 (IQR: 4–12.5), respectively (*p* = 0.001, *p* = 0.027, *p* = 0.004, and *p* = 0.041, respectively).

### The non-Nx group

The non-Nx group included 13 (44.8%) patients who were managed without nephrectomy. At the time CNS was diagnosed the median serum albumin and creatinine levels, and eGFR were 0.98 g/dL (IQR: 0.74–1.745), 0.17 mg/dL (IQR: 0.1–0.4), and 121.4 mL/min/1.73 m^2^ (IQR: 56–186), respectively. These values did not differ significantly from those in the Nx group (*p* = 0.53, *p* = 0.63, and *p* = 0.82, respectively). At the time of CNS diagnosis CKD staging was as follows: stage 1: *n* = 8 (66.7%); stage 2: *n* = 1 (8.3%); stage 3: *n* = 1 (8.3%); stage 4: *n* = 1 (8.3%); and stage 5: *n* = 1 (8.3%).

**Fig. 3:**
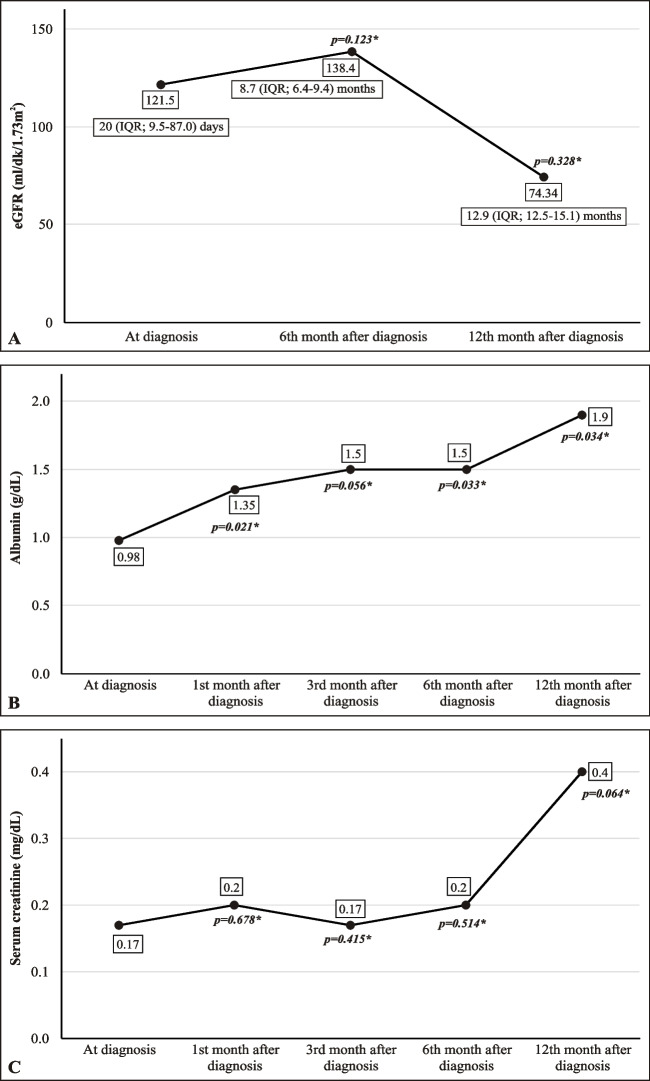
eGFR (**A**), the serum albumin level (**B**), and the serum creatinine level (**C**) before and after the diagnosis of CNS

During follow-up the median serum albumin level increased (*p* = 0.034), as compared to baseline, whereas the median serum creatinine level and eGFR did not change significantly (*p* = 0.4 and *p* = 0.32, respectively) (Fig. 3). At the time of CNS diagnosis, the median number of albumin infusions per month (day/month), and the median number of infections, hospitalizations per year, median hospitalization duration (days) were 12 (IQR: 8–24), 2 (IQR: 1–3.5), 4 (IQR: 2–5.75), and 9 (IQR: 2.5–10), respectively. By the 12 months post-CNS diagnosis the mean number of albumin infusions significantly decreased from 14.8 ± 8.7 to 9.1 ± 6.5 days/month, with values fluctuating at lower levels (from an IQR of 8–24 to IQR 3–15), whereas the median remained at 12 days/month (*p* = 0.007); however, the median number of infections (2 [IQR: 1–3.5], *p* = 0.589), hospitalizations (5 [IQR: [2–14], *p* = 0.5), and median hospitalization duration (days) (8 [IQR: 4–27.5]; *p* = 0.218), did not differ significantly from baseline.

### Comparison of the Nx and non-Nx groups

During follow-up the serum albumin and creatinine levels, and eGFR did not differ significantly between the 2 groups in the patients aged 1 and 2 years. ROC analysis showed that requiring albumin infusions ≥ 14 days/month predicted the decision to perform nephrectomy with 68% accuracy (73% sensitivity and 62% specificity). This threshold also corresponded to a negative predictive value of 67%, suggesting that patients requiring albumin infusions < 14 days/month could be safely managed without nephrectomy, as the present findings show nephrectomy was not required in any of the patients in the non-Nx group; however, height z-scores were higher in the non-Nx group in the patients aged 1 and 2 years (Table 1). Patient survival did not differ significantly between the 2 groups.

**Table 1:** Comparison of patients with and without nephrectomy during follow-up

Parameter	Nx group (n= 16) median (IQR)	Non-Nx group (n= 13) median (IQR)	p value
**At diagnosis**			
Serum albumin (g/dL)	1.34 (0.87–1.53)	0.98 (0.74–1.45)	0.52
Serum creatinine (mg/dL)	0.17 (0.1–0.3)	0.17 (0.1–0.4)	0.62
eGFR (ml/min/1.73 m²)	101.18 (70.2–371)	121.4 (56–186.8)	0.83
Length (cm)	49.5 (47.2–52.75)	47.5 (40.5–50)	0.16
Length z-score	− 2 [− 3-(− 1)]	− 2.5 (− 5-(− 1))	0.45
Weight (kg)	3.1 (2.8–3.7)	3 (2.12–3.75)	0.81
Weight z-score	− 2 [− 3-(− 1.5)]	− 1.5 (− 2.75-(− 1))	0.48
**At the first year**			
Serum albumin (g/dL)	1.8 (1.47–2.33)	1.85 (0.97–2.19)	0.24
Serum creatinine (mg/dL)	0.4 (0.19–1.22)	0.22 (0.12–0.64)	0.37
eGFR (ml/min/1.73 m²)	57 (22.5–159.5)	77.4 (35.7–341)	0.49
Length (cm)	68 (64.2–72.7)	73 (69.5–77.5)	0.005
Length z-score	− 2 [− 3.75-(− 1)]	− 1 (− 2- 0)	0.002
Weight (kg)	8 (6.7–8.8)	8.5 (7.45–10.2)	0.048
Weight z-score	− 1 [− 3.5-(− 1)]	− 1 (− 2.50- 0.25)	0.16
**At the second year**			
Serum albumin (g/dL)	2.55 (2–3.88)	2.1 (1.5–4.0)	0.41
Serum creatinine mg/dL	1.5 (0.3–3.6)	0.54 (0.4–1.7)	0.26
eGFR (ml/min/1.73 m²)	14.6 (8.6–104.4)	64.5 (18.0–91.3)	0.23
Length (cm)	79 (72.0–84)	81 (79.0–87,2)	0.19
Length z-score	− 2.5 (− 4-(− 1.75))	− 1 (− 2.5- 0)	0.11
Weight (kg)	9.5 (9–10)	11.5 (10.2–12.5)	0.056
Weight z-score	− 2 (− 2-(− 1))	− 1 (− 1- 0)	0.038

In patients with Nx, the median duration of follow-up of patients was 5.5 years (IQR: 2.2–8.75). At the time of the last follow-up visit the median serum albumin and creatinine levels, and eGFR were 3.7 g/dL (IQR: 1.9–4.2), 0.68 mg/dL (IQR: 0.5–2), and 62.8 mL/min/1.73 m^2^ (IQR: 14.7–106.3), respectively. The median body weight and height z-scores were –1.25 (IQR (–3.25)− 0)) and –2.25 (IQR:(–3)− 0)), respectively. During follow-up, 7 patients with Nx underwent kidney transplantation, whereas 3 patients were on peritoneal dialysis at the time of their last follow-up visit. The median age at the initiation of kidney replacement therapy was 2 years (IQR:1.7–2.9). Eleven patients experienced catheter-related complications, as follows: catheter-related infections: *n* = 6 (46.2%); thrombosis: *n* = 4 (30.8%); skin destruction: *n* = 1 (7.7%). During the follow-up period 2 patients with Nx died: 1 due to sepsis and 1 due to an unknown cause.

In patients with non-Nx, the median duration of follow-up was 9 years (IQR: 4.5–11). At the time of the last follow-up visit, the median serum albumin and serum creatinine levels, and eGFR were 4.3 g/dL (IQR: 3.6–4.6), 0.8 mg/dL (IQR: 0.6–1.6), and 61 mL/min/1.73 m^2^ (IQR: 19–111), respectively. During follow-up, 9 patients with non-Nx underwent kidney transplantation, whereas 1 patient was on peritoneal dialysis at the time of their last follow-up visit. The median age at the initiation of kidney replacement therapy was 2.9 years (IQR: 1.5–7.8). The median weight and height z-scores were –0.5 (IQR: (–2.12)− 1.13) and –1 (IQR:(–2)− 0)), respectively. During follow-up 5 patients experienced catheter-related infections. None of the patients had thrombosis. Additionally, 2 patients with non-Nx died during the follow-up period: 1 due to pneumonia and 1 due to an unknown cause following kidney transplantation.

## Discussion

The present findings show that nephrectomy significantly decreases the number of required albumin infusions, infections, and hospitalizations in patients with CNS secondary to *NPHS1* mutations. Furthermore, the study identified a novel threshold of requiring albumin infusions ≥ 14 days/month (as determined via ROC analysis) that predicts the need for nephrectomy with 73% sensitivity and 62% specificity, offering a practical tool for patient selection.

Nephrectomy has played a pivotal role in the management of CNS patients since 1980s. In the mid- 1980s infants with *NPHS1* mutations often succumbed to severe complications of CNS. To address this problem an aggressive management strategy adopted from the USA was implemented [1]. This approach involved regular albumin infusions until the patient reached a weight of approximately 7 kg (typically at age 6–10 months), followed by nephrectomy. Dialysis was then initiated as a bridge therapy until the patient was large enough to undergo kidney transplantation, usually at age 1–1.5 years. This aggressive treatment approach has been particularly challenging for most patients. Many spent much of their early life in hospital receiving frequent albumin infusions. Following the demanding nephrectomy surgery, families were required to learn and initiate peritoneal dialysis at home, a complex and stressful process associated with severe complications such as peritonitis and sepsis, which significantly increased morbidity and, in some cases, led to mortality [12]. As a result, an alternative conservative treatment strategy without nephrectomy was proposed, allowing patients to be discharged home for management [13]. Dufek et al. studied the outcome of this conservative strategy in 80 pediatric CNS patients from 17 nephrology departments [13]. Among this cohort, 55 patients had *NPHS1* mutations. Outcomes were compared between 2 groups of *NPHS1*-mutated patients: those that underwent bilateral nephrectomy and those managed conservatively. The incidence of septic or thrombotic episodes and growth outcomes did not differ significantly between the 2 groups. The response to antiproteinuric treatment, as well as kidney and patient survival, was independent of the type of *NPHS1* mutation. At the last follow-up visit (median age: 34 months), 20 (80%) patients in the nephrectomy group had undergone kidney transplantation, and 1 had died. In the conservative management group 53% of patients remained off dialysis, 4 (24%) patients underwent kidney transplantation, and 2 patients died. A French study further supported the viability of conservative treatment, reporting that patients could be weaned off albumin and discharged home, and that kidney survival did not differ significantly from that in patients who underwent nephrectomy [14]. The consensus recommendations of the ERKNet-ESPN Working Group suggest that an individualized, stepwise approach with prolonged conservative management is a viable alternative to early bilateral nephrectomy and dialysis; however, unilateral or bilateral nephrectomy can be considered in patients with severe complications such as failure to thrive and thrombosis, or difficulty maintaining intravascular euvolemia, despite optimized conservative treatment [2].

Currently, there is a lack of evidence-based data regarding the necessity and optimal timing of nephrectomy in CNS patients. The studies available in the literature are primarily based on collective analyses of patients sharing the CNS phenotype, but with various genetic abnormalities, including mutations in *NPHS1, NPHS2, WT1, LAMB2*, and *PLCE1* [2, 8, 9]; however, each genetic abnormality can result in a unique disease course and prognosis, necessitating individualized evaluation for optimal treatment planning [9]. To the best of our knowledge the present study includes the largest CNS cohort composed exclusively of individuals with bi-allelic *NPHS1* mutations. This study analyzed their clinical characteristics, prognoses, and the effect of nephrectomy on disease-associated morbidity and mortality.

The present findings show that the number of albumin infusions, infections, and hospitalizations decreased significantly after nephrectomy, as compared to the pre-nephrectomy period. In all, 13 (44.8%) of the present cohort were managed without nephrectomy. At the time CNS was diagnosed the median serum albumin and creatinine levels, and eGFR did not differ significantly different between the patients in the Nx and non-Nx groups. Among the patients in the non-Nx group, within a year, the serum albumin level increased compared to baseline, whereas the serum creatinine level and eGFR did not change significantly. Additionally, the body weight z-score increased in the non-Nx patients aged 1 and 2 years. Patient survival did not differ significantly between the Nx and non-Nx groups, which is consistent with earlier findings.

Suihko et al. [15] studied the effects of early nephrectomy (body weight 7 kg) versus late nephrectomy (body weight ≥ 10 kg), noting that infants who underwent late nephrectomy had comparable outcomes, with reduced feeding tube dependency and better overall quality of life than those who underwent early nephrectomy [12]. The present cohort aligns closely with that study’s early nephrectomy group, as the median age at the time of nephrectomy in the present study was 6.5 months (body weight approximately 7 kg). What remains unclear is which patients will benefit from nephrectomy. In the present study’s Nx group the median number of albumin infusions required was 20 days/month (IQR: 12–28), as compared to 12 days/month (IQR: 8–24) in the non-Nx group. Although the difference was not significant, it was likely due to the small number of patients in each group. Additional analysis yielded meaningful findings. For instance, ROC analysis showed that an albumin infusion requirement cut-off of 14 days/month predicted the decision to perform nephrectomy with 68% accuracy and a corresponding negative predictive value of 67%, indicating that patients requiring albumin infusion < 14 doses/month can likely be safely managed without nephrectomy. This novel finding might be considered for use as a predictive marker for identifying patients who would benefit from nephrectomy, offering valuable guidance for routine clinical practice.

Some clinicians opt for unilateral nephrectomy combined with the use of ACEi and/or non-steroidal anti-inflammatory drugs to decrease both the severity of proteinuria and the number of required albumin infusions [16]. In the present study, 13 patients in the Nx group underwent unilateral nephrectomy, versus 3 patients who underwent bilateral nephrectomy, of which 1 underwent bilateral nephrectomy at age 21 months and the other 2 (sequential nephrectomy patients) had their first nephrectomy at age 4 and 6 months, respectively. Recently, Murakoshi et al. [7] studied the efficacy of unilateral nephrectomy in a case series that included CNS patients with non-Fin major and non-Fin minor mutations. As in the present study, they observed a significant decrease in the mean albumin dose following nephrectomy (from 2.0 g/kg/day to 0.4 g/kg/day, *p* = 0.02) [7]. Even if nephrectomy does not alter the overall prognosis, its effectiveness in decreasing the number of albumin infusions required, infections, and hospitalizations may still have significant direct and indirect benefits. Regular and frequent albumin infusions often necessitate the placement of a central catheter, which is associated with inherent risks such as infection, bleeding, thrombosis, and the need for anticoagulant therapy. By decreasing the number of albumin infusions required, nephrectomy can eliminate the need for a central catheter, thereby mitigating catheter-related complications. Although the present study did not investigate the cost of treatment in the 2 groups, nephrectomy might also help decrease the overall cost of treatment.

This study has several limitations. First, its retrospective design may introduce inherent biases in data collection and clinical management. Second, as patients were recruited from multiple centers, variations in treatment protocols may have influenced certain outcomes. We used the number of albumin infusions as a comparative parameter; however, although infusion practices across centers were generally similar, they were not fully standardized. Moreover, the patients exhibited significant clinical and genetic variability, which limited the scope of certain statistical analyses. Lastly, comparison of the cost of treatment in the Nx and non-Nx groups was not performed.

In conclusion, nephrectomy is associated with a decrease in the number of albumin infusions required, infections, and hospitalizations, making it a potential treatment option for selected patients with CNS secondary to the *NPHS1* mutation. CNS patients with *NPHS1* mutations who require albumin infusions > 14 days/month, as well as those experiencing frequent infections and hospitalizations may benefit from nephrectomy; however, the decision to perform the procedure should be individually based on the clinical status in non-Fin major and non-Fin minor bi-allelic *NPHS1*-mutated patients.

## Supplementary information

Below is the link to the electronic supplementary material.

Graphical abstract (PPTX 254 KB)

## Data Availability

The dataset for this study is not publicly available due to concerns regarding participant/patient anonymity. Requests to access the dataset should be directed to the corresponding author.

## Code availability

Data subsets are available from the corresponding author upon reasonable request.
