# Publisher Correction: The clinical characteristics of patients with congenital nephrotic syndrome secondary to *NPHS1* mutation: Is nephrectomy still a therapeutic option for selected cases?

**DOI:** 10.1007/s00467-025-06806-1

**Published:** 2025-05-09

**Authors:** Yüksel Uğurlu, Bora Gülhan, İsmail Dursun, Hülya Nalçacıoğlu, Gülşah Kaya Aksoy, Nur Canpolat, Aysun Bayazıt, Zeynep Birsin Özçakar, Selcuk Yüksel, Gönül Parmaksız, Gülşah Özdemir, Eda Didem Kurt-Şükür, Ali Düzova, Mutlu Hayran, Fatih Ozaltin

**Affiliations:** 1 Department of Pediatrics, Faculty of Medicine, Hacettepe University, Ankara, Türkiye; 2 Department of Pediatric Nephrology, Faculty of Medicine, Hacettepe University, Sihhiye, 06100 Ankara, Türkiye; 3 Department of Pediatric Nephrology, Faculty of Medicine, Erciyes University, Kayseri, Türkiye; 4 Department of Pediatric Nephrology, Faculty of Medicine, Ondokuz Mayıs University, Samsun, Türkiye; 5 Department of Pediatric Nephrology, Faculty of Medicine, Akdeniz University, Antalya, Türkiye; 6 Department of Pediatric Nephrology, Istanbul University-Cerrahpasa, Cerrahpasa Faculty of Medicine, Istanbul, Türkiye; 7 Department of Pediatric Nephrology, Faculty of Medicine, Çukurova University, Adana, Türkiye; 8 Department of Pediatric Nephrology, Faculty of Medicine, Ankara University, Ankara, Türkiye; 9 Department of Pediatric Nephrology, Faculty of Medicine, Çanakkale Onsekiz Mart University, Çanakkale, Türkiye; 10 Department of Pediatric Nephrology, Başkent University Adana Dr. Turgut Noyan Training and Research Center, Adana, Türkiye; 11 Department of Preventive Oncology, Hacettepe University Cancer Institute, Ankara, Türkiye; 12 Nephrogenetics Laboratory, Department of Pediatric Nephrology, Faculty of Medicine, Hacettepe University, Ankara, Türkiye; 13 Center for Genomics and Rare Diseases, Hacettepe University, Ankara, Türkiye; 14 Department of Bioinformatics, Hacettepe University Institute of Health Sciences, Ankara, Türkiye

**Publisher Correction: Pediatric Nephrology**

10.1007/s00467-025-06774-6

The legend for Fig. 1 was previously omitted and has now been included as follows:

- "I: Nephrectomy; ✣: eGFR <60 ml/min/1.73 m2; ▿: Kidney transplantation; orange: albumin <2.5 g/dL; blue: albumin >2.5 g/dL; x: Exitus; three squares represent one year; Yes*: Sequential bilateral nephrectomy; Yes**: Concurrent bilateral nephrectomy."

The original article has been corrected, and the publisher apologizes for the inconvenience.

