# Supplementary material for: The clinical characteristics of patients with congenital nephrotic syndrome secondary to NPHS1 mutation: Is nephrectomy still a therapeutic option for selected cases?
Source: Pediatr Nephrol. 2025 Apr 23;40(8):2505–13. doi: 10.1007/s00467-025-06774-6 (PMC12187890; doi:10.1007/s00467-025-06774-6)
Supplement: Supplementary file 1 — Graphical abstract (PPTX 254 KB) [file 467_2025_6774_MOESM1_ESM.pptx]

## Slide 1
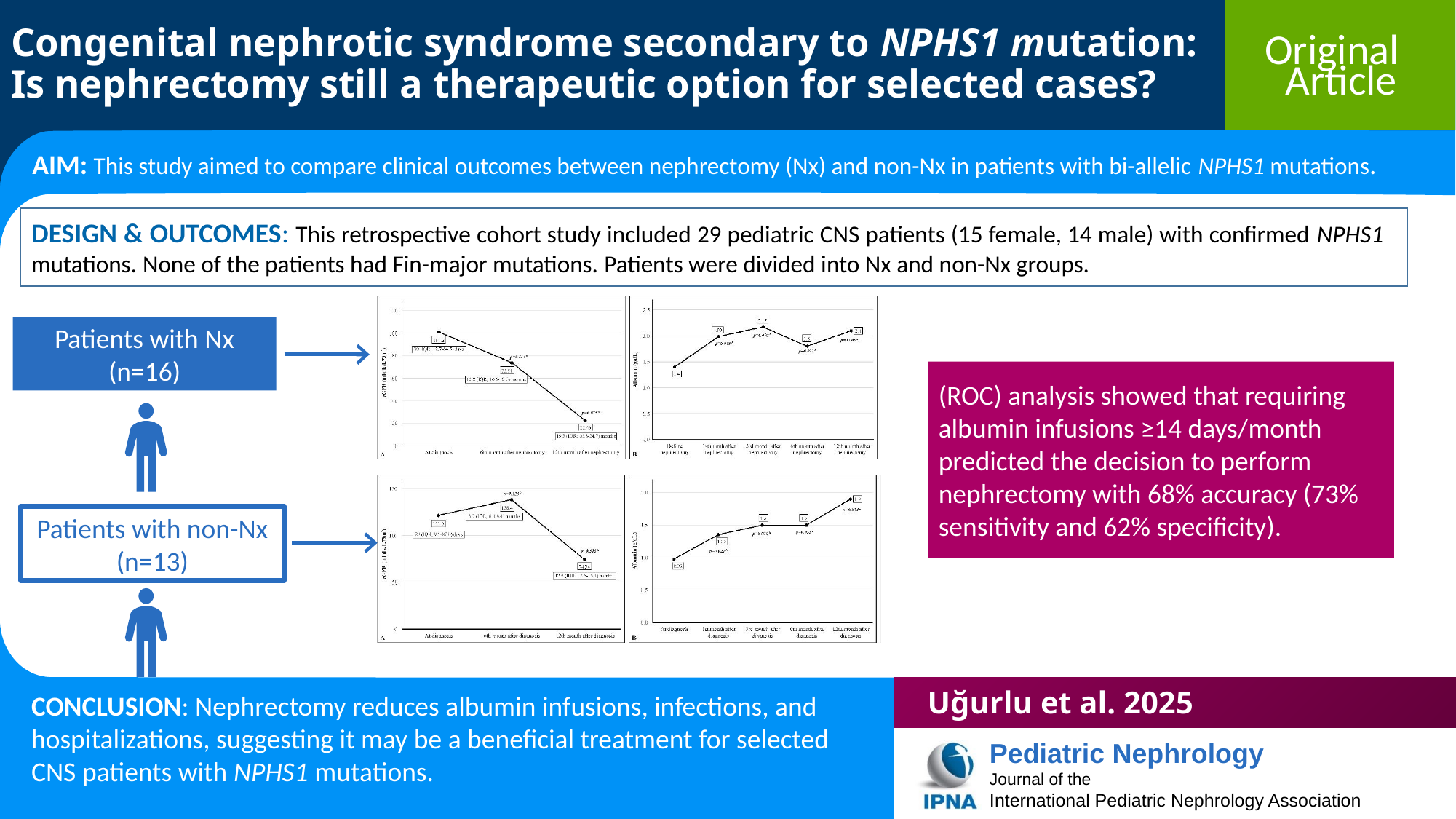

Congenital nephrotic syndrome secondary to NPHS1 mutation: Is nephrectomy still a therapeutic option for selected cases?
AIM: This study aimed to compare clinical outcomes between nephrectomy (Nx) and non-Nx in patients with bi-allelic NPHS1 mutations.
DESIGN & OUTCOMES: This retrospective cohort study included 29 pediatric CNS patients (15 female, 14 male) with confirmed NPHS1 mutations. None of the patients had Fin-major mutations. Patients were divided into Nx and non-Nx groups.
Patients with Nx (n=16)
(ROC) analysis showed that requiring albumin infusions ≥14 days/month predicted the decision to perform nephrectomy with 68% accuracy (73% sensitivity and 62% specificity).
Patients with non-Nx (n=13)
Uğurlu et al. 2025
CONCLUSION: Nephrectomy reduces albumin infusions, infections, and hospitalizations, suggesting it may be a beneficial treatment for selected CNS patients with NPHS1 mutations.
